# Association between depressive symptoms and employment type of Korean workers: the Fifth Korean Working Conditions Survey

**DOI:** 10.1186/s12889-023-17612-5

**Published:** 2024-01-04

**Authors:** Yun-Jung Yang, Jihye Lee

**Affiliations:** 1 Department of Convergence Science, College of Medicine, Catholic Kwandong University International St. Mary’s Hospital, Incheon, 22711 Republic of Korea; 2 Occupational Safety and Health Research Institute, Korea Occupational Safety and Health Agency, Ulsan, 44429 Republic of Korea

**Keywords:** Precarious worker, Depression, Korean working conditions survey, WHO-5 well-being index

## Abstract

**Background:**

This study analyzed the association between depressive symptoms and employment type, by considering both socioeconomic status and job stress factors.

**Methods:**

We analyzed 27,369 participants (13,134 men and 14,235 women) using data from the fifth Korean Working Conditions Survey. The participants were divided into regular and precarious workers. Depressive symptoms were defined using the World Health Organization-5 Well-Being Index. A multivariate logistic regression analysis was performed to assess the association between depressive symptoms and employment type.

**Results:**

Of the participants, 71.53% (*N* = 19578) were regular workers and 28.47% (*N* = 7791) were precarious workers. The weighted frequencies of participants with depressive symptoms (42.50%) were significantly higher than those of precarious workers (32.54%, *p* < 0.001). In the univariate and multivariate analyses, precarious workers had a significantly higher risk of depressive symptoms than regular workers (odds ratio [OR] 1.53, 95% confidence interval [CI] 1.42–1.64; OR 1.16, 95% CI 1.07–1.26, respectively). The significant association between depressive symptoms and precarious workers has also been reflected in propensity score matched participants through crude and multivariate analysis (OR 1.54 [95% CI 1.43–1.66] and OR 1.15 [95% CI 1.04–1.26], respectively).

**Conclusions:**

The findings suggest that precarious workers may have a higher risk of depressive symptoms than regular workers. However, this is only a cross-sectional study. Therefore, further study is required to investigate the relevance association between depressive symptoms and employment types.

**Supplementary Information:**

The online version contains supplementary material available at 10.1186/s12889-023-17612-5.

## Background

Over the past few decades, there has been a marked shift from standard to nonstandard employment (NSE) [1]. NSE is a grouping of different employment arrangements that deviate from the standard employment type, including temporary employment, part-time work, temporary agency work, other multi-party employment relationships, disguised employment relationships, and dependent self-employment. Another factor, precarious work, accounts for a considerable portion of NSE. While, to date, no internationally unified standard to define precarious work exists, it is broadly defined as workers who experience instability, lack of labor protection, insecurity, and social and/or economic vulnerability [2]. In Korea, it can be divided into non-permanent workers, part-time workers, and non-typical workers [3]. Companies in Korea have been actively using precarious workers because the labor market flexibility helps them reduce costs and manage manpower flexibly [4]. Previous study suggested that women and older people are more likely to be precarious worker [5]. Many women voluntarily resign or change to precarious jobs because of gender roles related to marriage or having children [6]. The proportion of precarious workers among all workers in Korea in 2022 will be 27.3%, the second highest among Organization for Economic Cooperation and Development (OECD) countries after the Netherlands (27.7%) [7].

Precarious work can be related to socioeconomic factors such as income, education level, marital status, job type, or social protection [6, 8]. Socioeconomic factors were identified as confounding factors in previous studies that analyzed precarious workers and depressive symptoms. In addition, several studies have reported that job stress is a harmful factor that increases the risk of depression [9–12]. To analyze the relationship between employment type and depressive symptoms, it is necessary to analyze job stress and socioeconomic status factors as well.

Mental disorders are one of the leading causes of global health-related burden, with depressive and anxiety disorders ranked among the top 25 [13, 14], according to the Global Burden of Diseases, Injuries, and Risk Factors Study (GBD) 2019.

It is important to identify and manage workers’ depressive symptoms, as the prevalence of such symptoms can impair their work [15]. One study evaluated an Employee Assistance Program and reported an 86% reduction in suicide risk among Japanese workers with preexisting suicidal thoughts [16]. These findings indicate the need for interventions to manage workers’ mental health problems such as depression.

To identify the relationship between precarious work and depressive symptoms, prospective data from the U.S. National Longitudinal Survey of Youth 1979 (NLSY79) of American men and women between 14 and 22 years of age in 1979 revealed that those who had been exposed to precarious work in the two years preceding the outcome measurement were at risk [17]. A study using the fifth Korea National Health and Nutrition Examination Survey data reported that precarious work was associated with depressive symptoms and suicidal ideation [18]. The Korean Welfare Panel Study indicated that employment status changes, from permanent to precarious work, were associated with the onset of depressive symptoms [6, 19]. Precarious work appeared to be associated with depressive symptoms after adjusting for socioeconomic factors; however, the evidence might be insufficient because the study subjects were relatively small and did not target workers.

We used data from The Korean Working Conditions Survey (KWCS) to evaluate the association between precarious work and depressive symptoms. The KWCS is one of the most representative surveys of Korean workers, with systematic and representative sampling of the Korean working population [20]. In this survey, depressive symptoms were investigated using the World Health Organization (WHO)-5 Well-Being Index. Although this index was designed as a tool to assess the quality of life and mood, it has sufficient validity as a screening tool for depressive symptoms as well and has been successfully applied in a wide range of research fields [21, 22]. However, studies using objective indicators such as the WHO-5 Well-Being Index on the association between precarious workers and depressive symptoms in a representative group in Korea are rare [6].

Therefore, this study aimed to analyze the association between depressive symptoms and the employment type of workers, considering both socioeconomic status and job stress factors, using an objective method with representative data from Korean workers.

## Methods

### Study population

The research data were taken from the fifth KWCS (2017), which was conducted by the Occupational Safety and Health Research Institute (OSHRI). The KWCS is a representative survey of Korean workers via a systematic and representative sampling of the Korean working population [20]. The KWCS was developed based on the European Working Conditions Survey (EWCS) conducted by the European Union (EU). The overall work environment was identified using a survey and included employment type, occupation, industry, exposure to risk factors, and employment stability. The KWCS uses the same questionnaire items as the EWCS, enabling the comparison with working conditions in European countries.

The KWCS has been conducted every four to five years since 2006 to determine risk factors by occupation and industry, and to identify the degree of exposure to risk factors by employment type. The population of the working environment survey is employed people (workers, business owners, and self-employed people) aged 15 or older in all households, and residing in Korea at the time of the survey. The survey subjects were extracted based on probability, proportional to size systematic sampling, that is, proportional to the number of households in the survey district. Weights were assigned to correct the difference between the distribution characteristics of the sample and the population. A total of 130,645 households were visited by researchers in the 5,000 survey districts sampled. Of these, 50,205 households were successfully interviewed and included in the final analysis. As such, the response rate of 50,205 people out of 130,645 people surveyed is 44.9%. The weight of the working environment survey was calculated by considering the design weight, response rate correction weight, and post-stratification correction weight. An investigator visited the survey subjects and conducted face-to-face interviews. The survey questionnaire assessed demographic characteristics, work environment, work characteristics, work organization, work hours, organizational communication, sociopsychological factors, health impact indicators, and job satisfaction.

**Fig. 1:**
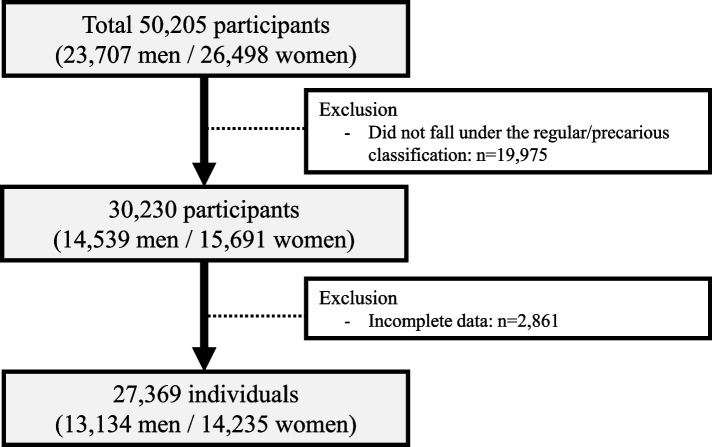
Flow diagram of the study population obtained from the fifth Korean working conditions survey (2015–2017)

The participants (*n* = 50,205) include workers, business owners, and self-employed individuals. According to the study’s purpose, subjects who were not regular or precarious workers were excluded (*n* = 19,975). We defined regular workers as those with an undetermined employment period, and non-regular workers as those with a fixed employment period [21]. Of the remaining subjects, those who did not respond to at least one question were excluded from the analysis (*n* = 2,861). Ultimately, 27,369 participants (13,134 men and 14,235 women) were included in the analysis (Fig. 1).

### Depressive symptoms

Depressive symptoms were estimated using the WHO-5 health index which showed the appropriate internal and external validity [23, 24]. The WHO-5 Well-Being Index was used to evaluate depressive symptoms in the fifth KWCS. Since its first publication in 1998, the WHO-5 has been translated into more than 30 languages and used in research studies worldwide [25]. The WHO-5 Well-Being Index has sufficient validity as a screening tool for depressive symptoms and as an outcome measure in clinical research, and has been successfully applied in a wide range of research fields [21, 22]. Thus, the WHO-5 Well-Being Index is an internationally comparable screening instrument.

Participants answered five questions about their feelings during the preceding two weeks. The items were as follows: “It was fun and I felt good,” “My mind was calm and comfortable,” “I was active and energetic,” “My fatigue disappeared and I awake and felt refreshed,” and “My daily life was full of interesting things.” The answers were answered on a six-point Likert scale of “always” (5 points), “mostly” (4 points), “more than half of the two weeks” (3 points), “less than half of the two weeks” (2 points), “sometimes” (1 points), and “never” (0 points). The total score for the five questions ranged from 0 to 25. A score above 13 was classified as normal, whereas a score of 13 or less was classified as a high risk for depression.

### Employment status

A questionnaire was used to ask participants about their current employment status. The KWCS classifies participants as workers, business owners, and self-employed workers. This study aimed to survey those who were employed, so it excluded self-employed, business owners, and unpaid family workers. The participants selected were classified as regular workers and precarious workers.

Precarious workers were defined as temporary workers, daily employed workers, and workers with a fixed working period. Additionally, participants with an undetermined employment period were categorized as regular workers. This classification was followed based on a previous study that explored the risk of employment types [26].

### Independent variables

#### Employment status

General and work-related characteristics of participants, including sex, age (15–19, 20–29, 30–39, 40–49, 50–59, or ≥ 60 years), education level (≤ elementary school, middle school, high school, or ≥ college), monthly income, living region (big city or small city), number of family members, self-rated health, working hours per week (≤ 40, 41–52, or ≥ 53 h), business size (1–9, 10–249, or ≥ 250 persons), working period at current job (year), shift work (yes or no), colleague support, and job satisfaction, were surveyed using face-to-face interviews. Based on the responses, monthly income was categorized as < 1 million won, 1–2 million won, 2–3 million won, or > 3 million won, and number of family members was categorized as single or two or more. The working period in the current job was classified by quartiles (lowest, lower-middle, upper-middle, and highest). Self-rated health was reclassified into two categories according to the questionnaire: very good and good (good) and fair, bad, or very bad (fair/poor). Colleagues support was reclassified as “always,” “most of the time,” or “sometimes yes” (yes); “not very much” or “not at all” (no); and “working alone” (not applicable). In addition, the responses to job satisfaction were reclassified into “yes” for “very satisfied” and “satisfied,” and “no” for “not very satisfied” and “not satisfied at all.”

### Statistical analysis

Since the KWCS to be used in this study employs a two-stage proportional stratified cluster sampling method, a complex sample analysis with weights applied was conducted to more accurately estimate the results of the population working environment survey. Weights are assigned in statistical surveys to increase the estimation accuracy, matching the structure of the population and sample structure using the difference in sampling rate according to sampling, response rate, and information about the population. The fifth KWCS provides a weighted variable for its data, representing the working population of Korea. To correct the difference between the distribution characteristics of the sample and population, the analysis was conducted using weights.

The means, standard deviations (continuous variables), and frequencies (categorical variables) were calculated for the regular and precarious groups. General and work-related characteristics were compared using Student’s t-test (continuous variables) or chi-squared test (categorical variables). A multivariate logistic regression analysis was performed to evaluate the relationship between employment status and depressive symptoms. The covariates of age, sex, education level, monthly income, region of residence, number of family members, self-rated health, working hours, business size, working period at the current job, shift work, colleague support, and job satisfaction were included in the regression model.

To adjust the unbalanced characteristics between regular and precarious workers, we also used a propensity score matching (PSM) analysis. Propensity score was estimated using a logistic regression model for each subject, based on age, sex, education level, monthly income, region of residence, number of family members, self-rated health, working hours, business size, working period at the current job, shift work, colleague support, and job satisfaction. We matched at a ratio of 1:1 between regular and precarious workers based on the estimated propensity score. The caliper width was 0.2 of the standard deviation of the logit-transformed propensity score. The univariate and multivariate analyses were also performed after PSM.

Data analyses were performed using STATA (version 16.0; StataCorp LP, College Station, TX, USA). Statistical significance at *p* < 0.05 was considered significant.

### Ethics statement

The KWCS is a government-approved survey of the National Statistics Office (No. 380002). It has been held every 3 years since 2006. The KWCS data used in this study are public, and personal information of all respondents is de-identified. The Institutional Review Board (IRB) of Catholic Kwandong University International St. Mary’s Hospital waived the requirement of written informed consent for participants (IS23EISI0014).

## Results

### General and work-related characteristics of the study population

The respondents were divided into two categories—regular and precarious—based on their employment status (Table 1). Of the 27,369 participants, 19,578 (71.53%) were regular workers and 7,791 (28.47%) were precarious workers. For regular workers the proportion of men (51.25%) and women (48.75%) was similar; however, for precarious workers, the proportion of women (60.20%) was higher than that of men (39.80%). Age, education level, monthly income, number of family members, and self-rated health were significantly different between regular and precarious workers (*p* < 0.001).

**Table 1:** Characteristics of the study subjects according to the employment status

	Total (*n* = 27,369)	Regular (*n* = 19,578)	Precarious (*n* = 7791)	*P*-value
Sex (n [%])				< 0.001
Men	13,134 (47.99)	10,033 (51.25)	3101 (39.80)	
Women	14,235 (52.01)	9545 (48.75)	4690 (60.20)	
Age (years; n [%])				< 0.001
15–19	223 (0.81)	62 (0.32)	161 (2.07)	
20–29	3613 (13.20)	2568 (13.12)	1.045 (13.41)	
30–39	6255 (22.85)	5288 (27.01)	967 (12.41)	
40–49	7029 (25.68)	5686 (29.04)	1343 (17.24)	
50–59	6271 (22.91)	4450 (22.73)	1821 (23.37)	
≥ 60	3978 (14.53)	1524 (7.78)	2454 (31.50)	
Education (n [%])				< 0.001
≤ Elementary school	1403 (5.13)	264 (1.35)	1139 (14.62)	
Middle school	1931 (7.06)	783 (4.00)	1148 (14.73)	
High school	9525 (34.80)	6211 (31.72)	3314 (42.54)	
≥ College	14,510 (53.02)	12,320 (62.93)	2190 (28.11)	
Income (million Korean Won; n [%]))				< 0.001
< 1	2619 (9.57)	416 (2.12)	2203 (28.28)	
1–1.99	8281 (30.26)	5025 (25.67)	3256 (41.79)	
2–2.99	8076 (29.51)	6593 (33.68)	1483 (19.03)	
≥ 3	8393 (30.63)	7544 (38.53)	849 (10.90)	
Region (n [%])				0.232
Big city	13,671 (49.95)	9824 (50.18)	3847 (49.38)	
Small city	13,698 (50.05)	9754 (49.82)	3944 (50.62)	
Household (n [%])				< 0.001
Single	5081 (18.56)	3109 (15.88)	1972 (25.31)	
2 or more	22,288 (81.44)	16,469 (84.12)	5819 (74.69)	
Self-rated health (n [%])				< 0.001
Good	19,807 (72.37)	14,931 (76.26)	4876 (62.59)	
Fair/poor	7562 (27.63)	4647 (23.74)	2915 (37.41)	

Data present the frequency (percent) as appropriate

In addition, the proportions of work-related characteristics, including working hours per week, business scale, working period at current job shift, colleague support, and job satisfaction, were significantly different between regular and precarious workers (all *p* < 0.001 (Table 2)).

**Table 2:** Work-related characteristics of the study subjects according to employment status

	Total (*n* = 27,369)	Regular (*n* = 19,578)	Precarious (*n* = 7791)	*P*-value
Working hours per week (n [%])				< 0.001
≤ 40	15,995 (58.44)	10,899 (55.67)	5096 (65.41)	
41–52	7498 (27.40)	5898 (30.13)	1600 (20.54)	
≥ 53	3876 (14.16)	2781 (14.20)	1095 (14.05)	
Business scale (n [%])				< 0.001
1–9	12,122 (44.29)	7739 (39.53)	4383 (56.26)	
10–249	13,105 (47.88)	9978 (50.97)	3127 (40.14)	
≥ 250	2142 (7.83)	1861 (9.51)	281 (3.61)	
Working period at current job (year)	6.48 ± 6.82	7.52 ± 6.94	3.89 ± 5.75	< 0.001
Shift work (n [%])				< 0.001
Yes	3363 (12.29)	2159 (11.03)	1204 (15.45)	
No	24,006 (87.71)	17,419 (88.97)	6587 (84.55)	
Colleague support (n [%])				< 0.001
Yes	24,038 (87.83)	17,867 (91.26)	6171 (79.21)	
No	1359 (4.97)	784 (4.00)	575 (7.38)	
Not applicable	1972 (7.21)	927 (4.73)	1045 (13.41)	
Job satisfaction (n [%])				< 0.001
Yes	21,078 (77.01)	15,702 (80.20)	5376 (69.00)	
No	6291 (2299)	3876 (19.80)	2415 (31.00)	

Data present the mean ± standard deviations or frequency (percent) as appropriate

### Depressive symptoms by employment status

The prevalence of depressive symptoms in regular and precarious workers was evaluated using the WHO-5 scores (Table 3). The unweighted frequencies (%) of depressive symptoms were 33.43% for regular workers and 44.29% for precarious workers (*p* < 0.001). The weighted frequency (%) of depressive symptoms was also significantly higher in precarious workers (42.50%) than in regular workers (32.54%; *p* < 0.001).

**Table 3:** Prevalence of depressive symptoms in the study subjects according to employment status

	WHO-5 well-being index	Regular (*n* = 19,578)	Precarious (*n* = 7791)	*P*-value
Unweighted (*n* = 27,369)	Normal (> 13)	13,034 (66.57)	4340 (55.71)	< 0.001
	Depressive symptom (≤ 13)	6544 (33.43)	3451 (44.29)	
Weighted (*n* = 18,164,016)	Normal (> 13)	9,247,680 (67.46)	2,561,468 (57.50)	< 0.001
	Depressive symptom (≤ 13)	4,461,458 (32.54)	1,893,410 (42.50)	

*WHO* World Health Organization

### The association between depressive symptoms and employment status

Odds ratios (ORs) and 95% confidence intervals (CIs) for depressive symptoms based on WHO-5 scores according to employment status are presented in Table 4. Regular workers were considered the reference group in the multivariate logistic analysis. Crude and multivariate analyses revealed that precarious workers had significantly increased depressive symptoms compared with regular workers (all *p* < 0.001). Even after adjusting for weight, it was maintained that precarious workers had a significantly higher risk of depression than regular workers (all *p* < 0.001).

**Table 4:** Association between depressive symptoms and employment status in the study population

	Crude		Model1		Model2	
	OR (95% CI)	*P*-value	OR (95% CI)	*P*-value	OR (95% CI)	*P*-value
Unweighted						
Regular	1.00		1.00		1.00	
Precarious	1.58 (1.50–1.67)	< 0.001	1.23 (1.16–1.31)	< 0.001	1.15 (1.08–1.23)	< 0.001
Weighted						
Regular	1.00		1.00		1.00	
Precarious	1.53 (1.42–1.64)	< 0.001	1.25 (1.15–1.36)	< 0.001	1.16 (1.07–1.26)	< 0.001

*OR* odds ratio, *CI* confidence interval Model 1: Crude + sex, age, education, income, and area. Model 2: Model 1 + number of households, health status, working hours, business scale, working period at current job, colleague support, and job satisfaction

### Propensity score matching (PSM) analysis

After PSM adjustment, 11,152 participants were included to assess the relationship between depressive symptoms and employment type. The participants’ general and work-related characteristics are presented in Tables S2 and S3. The number of depressive symptoms, based on the WHO questionnaire, was significantly higher in precarious workers compared to those experienced by regular workers (Table S4).

In crude analysis, depressive symptoms in precarious workers had significantly higher OR compared to that of regular workers (OR 1.54 [95% CI 1.43–1.66], Table 5). After adjustment with general characteristics (Model 1), the association of depressive symptoms in precarious workers was significantly higher than in regular workers (OR 1.24 [95% CI 1.13–1.36]). On further adjustment with all covariates (Model 2), OR in the precarious workers was higher than that in regular workers (1.15 [95% CI 1.04–1.26]).

**Table 5:** Association between depressive symptoms and employment status in the study population after propensity score matching

	Crude		Model1		Model2	
	OR (95% CI)	*P*-value	OR (95% CI)	*P*-value	OR (95% CI)	*P*-value
Regular	1.00		1.00		1.00	
Precarious	1.54 (1.43–1.66)	< 0.001	1.24 (1.13–1.36)	< 0.001	1.15 (1.04–1.26)	0.003

*OR* odds ratio, *CI* confidence interval Model 1: Crude + sex, age, education, income, and area Model 2: Model 1 + number of households, health status, working hours, business scale, working period at current job, colleague support, and job satisfaction

## Discussion

This study evaluated the association between depressive symptoms and employment type in Korean workers using the fifth KWCS. In this study, the unweighted frequencies (%) of depressive symptoms were 33.43% for regular workers and 44.29% for precarious workers (*p* < 0.001) (Table 3). The frequency of depressive symptoms was 42.50% in precarious workers and 32.54% in regular workers (*p* < 0.001). The prevalence of depressive symptoms was relatively higher than that reported in the previous study, in which depressive mood was reported to be 13.1% for precarious workers and 7.8% for regular workers [16]. This might be due to the difference in the definition of precarious workers, as well as the assessment method for diagnosis of depressive symptoms.

Crude and multivariate analyses showed that precarious workers had significantly increased depressive symptoms compared with regular workers (all *p* < 0.001, Table 4). The precarious workers had significantly higher risks of depression than regular workers after adjusting weights and other variables (OR 1.16; 95% CI 1.07–1.16). After PSM adjustment, the significant association between depressive symptoms and precarious workers remained in both crude and multivariate analyses (Table 5). In a Korean cross-sectional study, the adjusted OR for depressive symptoms in precarious workers was 1.32 [18]. Another recent study on the Korean population suggested the independent effects of precarious work on depression after controlling for confounding factors [6]. One study showed that the transition from full-time permanent employment to another employment status is associated with the development of severe depressive symptoms [8]. A study using nationally representative data from the United States also found that exposure to precarious work was associated with increased depressive symptom severity [17]. A study using nationally representative data from Chile also showed that precarious work was associated with anxiety and depression after controlling for age, sex, and occupational group [27]. A Swedish study suggested that precarious employment is a risk factor for poor mental health among young individuals [28]. The independent effects of precarious work on poor mental health status have been consistently suggested in previous studies, and with regard to the mechanism of this association, the possible influence of psychological stress induced by job insecurity or disadvantaged job conditions on workers’ mental health has been suggested [29]. As an explanation for the association, people with poor mental health in the early stages of life are more likely to have precarious work, as their mental health problems can potentially reduce their chances of obtaining a quality job [30]. Depressive symptoms and job instability may have a vicious cycle.

We found that socioeconomic factors, such as age, gender, education, income, and current health status were associated with both precarious work and depressive symptoms in adult workers (Supplementary Table S1). In this study, the adjusted OR for depressive symptoms of women was 0.91 (95% CI 0.84–0.98). This was consistent with a previous study of Japanese workers in which the relationship between precarious work and severe psychological distress was significant only for male workers in the adjusted model [29]. A previous study also indicated that employment changes from regular to precarious work were associated with the onset of new depressive symptoms for the families’ main earners [8]. Our results are explained by the fact that the most common earners in a Japanese or South Korean worker’s family are likely to be men. We also find that lack of job satisfaction and support from colleagues affected depressive symptoms (OR 1.27, 95% CI 1.09–1.47; OR 1.67, 95% CI 1.55–1.80). Job satisfaction is associated with the incidence of burnout symptoms at work and can affect depressive symptoms [31]. Moreover, one study found that low control over worktime was associated with increased depression/anxiety scores (relative risk [RR] 1.61, 95% confidence interval [CI] 1.39–1.87) [27]. These results demonstrate the need for job stress interventions in the workplace.

This study had some limitations. First, it was a cross-sectional study. Although precarious work and depressive symptoms are related, it is not possible to establish a causality between the two. Longitudinal studies, such as cohort studies, may be helpful for this purpose. Second, factors related to depressive symptoms, which may include a person’s history of psychological diseases such as major depressive disorder and anxiety disorder, were not considered. We attempted to compensate for this limitation by adding a self-rated health item to our questionnaire. In addition, although smoking and alcohol consumption might be linked to depressive symptoms [32], the fifth KWCS does not survey these factors. Smoking and alcohol consumption were investigated by up to the fourth KWCS; however, due to the inaccurate responses in the self-reported questionnaire, these were excluded from fifth KWCS. Therefore, unfortunately, we were unable to adjust for smoking and alcohol consumption in this study. As such, a more accurate assessment of smoking and alcohol consumption in future surveys may help assess the risk of workers developing symptoms of depression.

Third, only workers currently employed at the time of the survey were included in the KWCS. Workers who already had severe depression, took a break from work, or quit treatment completely were excluded.

Despite these limitations, this study analyzed depressive symptoms according to one’s employment type using a questionnaire and representative data from Korean workers by applying weights and analyzing socioeconomic and job stress factors. Future research should analyze various occupational characteristics, such as industry classification, subtype of precarious work, and work type according to the WHO-5 Well-Being Index. These studies may provide important basic data for preventing and managing depression among workers.

### Supplementary Information

**Additional file 1: Table S1.** Association between general and depressive symptom-related characteristics and employment status in the study population, using multivariate logistic regression analysis. **Table S2.** Characteristics of the study subjects after propensity score matching according to the employment status. **Table S3.** Work-related characteristics of the study subjects after propensity score matching according to employment status. **Table S4.** Prevalence of depressive symptoms in the study subjects after propensity score matching according to employment status.

## Data Availability

The datasets used and/or analyzed in the current study are available here: https://oshri.kosha.or.kr/oshri/researchField/workingEnvironmentSurvey.do

## Abbreviations

EWCS: The European Working Conditions Survey
GBD: Global Burden of Diseases
KWCS: The Korean Working Conditions Survey
NSE: Non-standard employment
OECD: Organization for Economic Cooperation and Development
WHO: World Health Organization

## References

[1] ILO (2016). Non-standard employment around the world: understanding challenges, shaping prospects.

[2] Famira-Muehlberger U, Michalos AC (2014). Precarious Work. Encyclopedia of quality of life and well-being research.

[3] Jang S (2012). A study on the definition of non-regular workers and its scale in Korea. Korean J Ind Relat.

[4] Chung D-B (2018). The relationship between regular/irregular workers and job satisfaction - the comparison of group differences among young workers. Q J Lab Policy.

[5] Kim WY (2014). The size of nonregular workers and the analysis of its recent trend. J Labour Economics.

[6] Yoo KB, Park EC, Jang SY, Kwon JA, Kim SJ, Cho KH (2016). Association between employment status change and depression in Korean adults. BMJ Open.

[7] OECD. Temporary employment (2022). https://data.oecd.org/emp/temporary-employment.htm. Accessed 11 Jul 2023

[8] Jang SY, Jang SI, Bae HC, Shin J, Park EC (2015). Precarious employment and new-onset severe depressive symptoms: a population-based prospective study in South Korea. Scand J Work Environ Health.

[9] Kawakami N, Haratani T, Araki S (1992). Effects of perceived job stress on depressive symptoms in blue-collar workers of an electrical factory in Japan. Scand J Work Environ Health.

[10] Melchior M, Caspi A, Milne BJ, Danese A, Poulton R, Moffitt TE (2007). Work stress precipitates depression and anxiety in young, working women and men. Psychol Med.

[11] Oshio T, Tsutsumi A, Inoue A (2015). Do time-invariant confounders explain away the association between job stress and workers' mental health? Evidence from Japanese occupational panel data. Soc Sci Med.

[12] Wang J, Schmitz N, Dewa C, Stansfeld S (2009). Changes in perceived job strain and the risk of major depression: results from a population-based longitudinal study. Am J Epidemiol.

[13] GBD 2019 Diseases and Injuries Collaborators (2020). Global burden of 369 diseases and injuries in 204 countries and territories, 1990–2019: a systematic analysis for the global burden of disease study 2019. Lancet..

[14] GBD 2019 Mental Disorders Collaborators (2022). Global, regional, and national burden of 12 mental disorders in 204 countries and territories, 1990–2019: a systematic analysis for the Global Burden of Disease Study 2019. Lancet Psychiatry..

[15] Enns MW, Bernstein CN, Kroeker K, Graff L, Walker JR, Lix LM (2018). The association of fatigue, pain, depression and anxiety with work and activity impairment in immune mediated inflammatory diseases. PLoS ONE.

[16] Nakao M, Nishikitani M, Shima S, Yano E (2007). A 2-year cohort study on the impact of an Employee Assistance Programme (EAP) on depression and suicidal thoughts in male Japanese workers. Int Arch Occup Environ Health.

[17] Quesnel-Vallée A, DeHaney S, Ciampi A (2010). Temporary work and depressive symptoms: a propensity score analysis. Soc Sci Med.

[18] Han KM, Chang J, Won E, Lee MS, Ham BJ (2017). Precarious employment associated with depressive symptoms and suicidal ideation in adult wage workers. J Affect Disord.

[19] Kim SS, Subramanian SV, Sorensen G, Perry MJ, Christiani DC (2012). Association between change in employment status and new-onset depressive symptoms in South Korea - a gender analysis. Scand J Work Environ Health.

[20] Kim HY, Choi J, Lim HM, Park C, Hong YC (2021). The association between non-regular work patterns and insomnia among Korean wage workers: the fifth Korean working condition survey. Ann Occup Environ Med.

[21] Topp CW, Østergaard SD, Søndergaard S, Bech P (2015). The WHO-5 well-being index: a systematic review of the literature. Psychother Psychosom.

[22] Holm-Hadulla RM, Klimov M, Juche T, Möltner A, Herpertz SC (2021). Well-being and mental health of students during the COVID-19 pandemic. Psychopathology.

[23] Bonsignore M, Barkow K, Jessen F, Heun R (2001). Validity of the five-item WHO Well-Being Index (WHO-5) in an elderly population. Eur Arch Psychiatry Clin Neurosci..

[24] Krieger T, Zimmermann J, Huffziger S, Ubl B, Diener C, Kuehner C (2014). Measuring depression with a well-being index: further evidence for the validity of the WHO Well-Being Index (WHO-5) as a measure of the severity of depression. J Affect Disord.

[25] World Health Organization. Wellbeing measures in primary health care/the DepCare Project: report on a WHO meeting: Stockholm, Sweden. In: World Health Organization. Regional Office for Europe**;** 1998:p. 39.

[26] Lee J, An J (2016). A study on risk-exposure degree in working conditions: comparative analysis by employment type. Korea J Ind Relat.

[27] Lopez G, Kriebel D, Cifuentes M, Quinn M (2021). Effects of precarious work on symptomatology of anxiety and depression in Chilean workers, a cross sectional study. BMC Public Health.

[28] Canivet C, Bodin T, Emmelin M, Toivanen S, Moghaddassi M, Östergren PO (2016). Precarious employment is a risk factor for poor mental health in young individuals in Sweden: a cohort study with multiple follow-ups. BMC Public Health.

[29] Kachi Y, Inoue M, Nishikitani M, Yano E (2014). Differences in self-rated health by employment contract and household structure among Japanese employees: a nationwide cross-sectional study. J Occup Health.

[30] Virtanen M, Kivimaki M, Joensun M, Virtanen P, Elovainio M, Vahtera J (2005). Temporary employment and health: a review. Int J Epidemiol.

[31] Myhren H, Ekeberg O, Stokland O (2013). Job satisfaction and burnout among intensive care unit nurses and physicians. Crit Care Res Pract.

[32] Çelik N, Ceylan B, Ünsal A, Çağan Ö (2019). Depression in health college students: relationship factors and sleep quality. Psychol Health Med.

