# Supplementary material for: Association between depressive symptoms and employment type of Korean workers: the Fifth Korean Working Conditions Survey
Source: BMC Public Health. 2024 Jan 4;24:93. doi: 10.1186/s12889-023-17612-5 (PMC10768360; doi:10.1186/s12889-023-17612-5)
Supplement: Supplementary file 1 — Additional file 1: Table S1. Association between general and depressive symptom-related characteristics and employment status in the study population, using multivariate logistic regression analysis. Table S2. Characteristics of the study subjects after propensity score matching according to the employment status. Table S3. Work-related characteristics of the study subjects after propensity score matching according to employment status. Table S4. Prevalence of depressive symptoms in the study subjects after propensity score matching according to employment status. [file 12889_2023_17612_MOESM1_ESM.docx]

Table S1. Association between general and depressive symptom-related characteristics and employment status in the study population, using multivariate logistic regression analysis

|  | Unweighted (n=27,369) | | Weighted (n=18,164,016) | |
| --- | --- | --- | --- | --- |
|  | OR (95% CI) | P-value | OR (95% CI) | P-value |
| Sex |  |  |  |  |
| Men | 1.00 |  | 1.00 |  |
| Women | 0.94 (0.88–0.99) | 0.041 | 0.91 (0.84–0.98) | 0.020 |
| Age (years) |  |  |  |  |
| 15–19 | 1.00 |  | 1.00 |  |
| 20–29 | 1.80 (1.28–2.52) | 0.001 | 1.99 (1.30–3.04) | 0.001 |
| 30–39 | 2.32 (1.65–3.24) | <0.001 | 2.47 (1.62–3.77) | <0.001 |
| 40–49 | 2.40 (1.72–3.36) | <0.001 | 2.51 (1.64–3.82) | <0.001 |
| 50–59 | 2.43 (1.74–3.40) | <0.001 | 2.51 (1.65–3.83) | <0.001 |
| ≥60 | 2.61 (1.86–3.66) | <0.001 | 2.58 (1.68–3.95) | <0.001 |
| Education |  |  |  |  |
| ≤Elementary school | 1.00 |  | 1.00 |  |
| Middle school | 0.85 (0.73–0.99) | 0.040 | 0.82 (0.67–1.00) | 0.050 |
| High school | 0.82 (0.71–0.95) | 0.008 | 0.78 (0.64–0.93) | 0.008 |
| ≥College | 0.72 (0.62–0.84) | <0.001 | 0.67 (0.55–0.81) | <0.001 |
| Income (million KRW) |  |  |  |  |
| <1 | 1.00 |  | 1.00 |  |
| 1–1.99 | 0.91 (0.82–1.01) | 0.094 | 0.95 (0.83–1.10) | 0.558 |
| 2–2.99 | 0.82 (0.73–0.92) | 0.001 | 0.79 (0.68–0.92) | 0.003 |
| ≥3 | 0.76 (0.67–0.87) | <0.001 | 0.75 (0.64–0.89) | 0.001 |
| Region |  |  |  |  |
| Big city | 1.00 |  | 1.00 |  |
| Small city | 1.06 (1.01–1.11) | 0.019 | 0.96 (0.90–1.02) | 0.277 |
| Household |  |  |  |  |
| Single | 1.00 |  | 1.00 |  |
| 2 or more | 1.07 (0.99–1.14) | 0.052 | 1.09 (1.00–1.19) | 0.031 |
| Self-rated health |  |  |  |  |
| Good | 1.00 |  | 1.00 |  |
| Fair/poor | 2.00 (1.88–2.11) | <0.001 | 2.04 (1.89–2.19) | <0.001 |
| Working hours per week |  |  |  |  |
| ≤40 | 1.00 |  | 1.00 |  |
| 41–52 | 1.01 (0.95–1.08) | 0.547 | 1.07 (0.99–1.16) | 0.060 |
| ≥53 | 1.28 (1.18–1.38) | <0.001 | 1.29 (1.17–1.43) | <0.001 |
| Business scale |  |  |  |  |
| 1–9 | 1.00 |  | 1.00 |  |
| 10–249 | 1.09 (1.03–1.15) | 0.002 | 1.16 (1.08–1.25) | <0.001 |
| ≥250 | 1.16 (1.04–1.29) | 0.006 | 1.25 (1.10–1.42) | 0.001 |
| Working period at current job (year) | 0.99 (0.99–1.00) | 0.323 | 0.99 (0.99–1.00) | 0.506 |
| Shift work |  |  |  |  |
| Yes | 1.00 |  | 1.00 |  |
| No | 1.02 (0.94–1.11) | 0.509 | 1.01 (0.91–1.12) | 0.770 |
| Colleague support |  |  |  |  |
| Yes | 1.00 |  | 1.00 |  |
| No | 1.36 (1.21–1.53) | <0.001 | 1.27 (1.09–1.47) | 0.001 |
| Not applicable | 1.35 (1.22–1.49) | <0.001 | 1.42 (1.25–1.61) | <0.001 |
| Job satisfaction |  |  |  |  |
| Yes | 1.00 |  | 1.00 |  |
| No | 1.68 (1.58–1.79) | <0.001 | 1.67 (1.55–1.80) | <0.001 |

OR: odds ratio; CI: confidence interval; KRW: Korean won

Table S2. Characteristics of the study subjects after propensity score matching according to the employment status

|  | Total  (n=11152) | | | Regular  (n=5576) | | | Precarious  (n=5576) | | | P-value |
| --- | --- | --- | --- | --- | --- | --- | --- | --- | --- | --- |
| Sex (n [%]) |  | | |  | | |  | | | <0.001 |
| Men | 5092 (45.66) | | | 2864 (51.36) | | | 2228 (39.96) | | |  |
| Women | 6060 (54.34) | | | 2712 (48.64) | | | 3348 (60.04) | | |  |
| Age (years; n [%]) |  | | |  | | |  | | | <0.001 |
| 15–19 | 139 (1.25) | | | 20 (0.36) | | | 119 (2.13) | | |  |
| 20–29 | 1475 (13.23) | | | 713 (12.79) | | | 762 (13.67) | | |  |
| 30–39 | 2257 (20.24) | | | 1552 (27.83) | | | 705 (12.64) | | |  |
| 40–49 | 2569 (23.04) | | | 1605 (28.78) | | | 964 (17.29) | | |  |
| 50–59 | 2518 (22.58) | | | 1252 (22.45) | | | 1266 (22.70) | | |  |
| ≥60 | 2194 (19.67) | | | 434 (7.78) | | | 1760 (31.56) | | |  |
| Education (n [%]) |  | | |  | | |  | | | <0.001 |
| ≤Elementary school | 896 (8.03) | | | 80 (1.43) | | | 816 (14.63) | | |  |
| Middle school | 1027 (9.21) | | | 228 (4.09) | | | 799 (14.33) | | |  |
| High school | 4114 (36.89) | | | 1723 (30.90) | | | 2391 (42.88) | | |  |
| ≥College | 5115 (45.87) | | | 3545 (63.58) | | | 1570 (28.16) | | |  |
| Income (million KRW, n [%])) | |  | | |  | | | <0.001 | | |
| <1 | 1714 (15.37) | | | 127 (2.28) | | | 1587 (28.46) | | |  |
| 1–1.99 | 3756 (33.68) | | | 1444 (25.90) | | | 2312 (41.46) | | |  |
| 2–2.99 | 2897 (25.98) | | | 1834 (32.89) | | | 1063 (19.06) | | |  |
| ≥3 | 2785 (24.97) | | | 2171 (38.93) | | | 614 (11.01) | | |  |
| Region (n [%]) |  | | |  | | |  | | | 0.043 |
| Big city | 5579 (50.03) | | | 2843 (50.99) | | | 2736 (49.07) | | |  |
| Small city | 5573 (49.97) | | | 2733 (49.01) | | | 2840 (50.93) | | |  |
| Household (n [%]) |  | | |  | | |  | | | <0.001 |
| Single | 2339 (20.97) | | | 920 (16.50) | | | 1419 (25.45) | | |  |
| 2 or more | 8813 (79.03) | | | 4656 (83.50) | | | 4157 (74.55) | | |  |
| Self-rated health (n [%]) | | |  | | |  | | | <0.001 | |
| Good | 7752 (69.51) | | | 4231 (75.88) | | | 3521 (63.15) | | |  |
| Fair/poor | 3400 (30.49) | | | 1345 (24.12) | | | 2055 (36.85) | | |  |

KRW: Korean won. Data present the frequency (percent) as appropriate.

Table S3. Work-related characteristics of the study subjects after propensity score matching according to employment status

|  | Total  (n=11152) | Regular  (n=5576) | Precarious  (n=5576) | P-value |
| --- | --- | --- | --- | --- |
| Working hours per week (n [%]) |  |  |  | <0.001 |
| ≤40 | 6731 (60.36) | 3089 (55.40) | 3642 (65.32) |  |
| 41–52 | 2822 (25.30) | 1695 (30.40) | 1127 (20.21) |  |
| ≥53 | 1599 (14.34) | 792 (14.20) | 807 (14.47) |  |
| Business scale (n [%]) |  |  |  | 0.087 |
| 1–9 | 5374 (48.19) | 2233 (40.05) | 3141 (56.33) |  |
| 10–249 | 5048 (45.27) | 2811 (50.41) | 2237 (40.12) |  |
| ≥250 | 730 (6.55) | 532 (9.54) | 198 (3.55) |  |
| Working period at current job (year) | 5.65±6.58 | 7.43±6.83 | 3.87±5.80 | <0.001 |
| Shift work (n [%]) |  |  |  | <0.001 |
| Yes | 1486 (13.32) | 616 (11.05) | 870 (15.60) |  |
| No | 9666 (86.68) | 4960 (88.95) | 4706 (84.40) |  |
| Colleague support (n [%]) |  |  |  | <0.001 |
| Yes | 9469 (84.91) | 5051 (90.58) | 4418 (79.23) |  |
| No | 635 (5.69) | 244 (4.38) | 391 (7.01) |  |
| Not applicable | 1048 (9.40) | 281 (5.04) | 767 (13.76) |  |
| Job satisfaction (n [%]) |  |  |  | <0.001 |
| Yes | 8372 (75.07) | 4540 (81.42) | 3832 (68.72) |  |
| No | 2780 (24.93) | 1036 (18.58) | 1744 (31.28) |  |

Data are shown as the mean ± standard deviations or frequency (percent) as appropriate.

Table S4. Prevalence of depressive symptoms in the study subjects after propensity score matching according to employment status

| WHO-5 well-being index | Regular  (n=5576) | Precarious  (n=5576) | P-value |
| --- | --- | --- | --- |
| Normal (>13) | 3680 (66.00) | 3105 (55.69) | <0.001 |
| Depressive symptom (≤13) | 1896 (34.00) | 2471 (44.31) |  |

WHO: World Health Organization
